# A phase II study of chidamide, cytarabine, aclarubicin, granulocyte colony-stimulating factor, and donor lymphocyte infusion for relapsed acute myeloid leukemia and myelodysplastic syndrome after allogeneic hematopoietic stem cell transplantation

**DOI:** 10.1007/s12032-022-01911-9

**Published:** 2023-01-10

**Authors:** Yan Wei, Lijun Wang, Chengying Zhu, Honghua Li, Jian Bo, Ran Zhang, Ning Lu, Yongli Wu, Xiaoning Gao, Liping Dou, Daihong Liu, Chunji Gao

**Affiliations:** 1 Medical School of Chinese PLA, Beijing, China; 2 Department of Hematology, The Fifth Medical Centre, Chinese PLA General Hospital, Beijing, 100071 China; 3 School of Medicine, Nankai University, Tianjin, China

**Keywords:** Acute myeloid leukemia, CAG regimen, Chidamide, Myelodysplastic syndrome, Relapse

## Abstract

Chemotherapy followed by donor lymphocyte infusion (DLI) is a promising treatment for relapsed acute myeloid leukemia (AML) and myelodysplastic syndrome (MDS) after allogeneic hematopoietic stem cell transplantation (allo-HSCT). However, the best strategy for administering this therapy is still unclear. This study sought to explore the efficacy and safety of chidamide and CAG (cytarabine, aclarubicin, and granulocyte colony-stimulating factor) (CCAG) regimen followed by DLI in relapsed AML/MDS after allo-HSCT. This was a single-arm, phase II trial in patients with relapsed AML/MDS after allo-HSCT. CCAG regimen followed by DLI was given according to the inclusion and exclusion criteria. Twenty adult patients were enrolled. The median follow-up time was 12 months. The complete remission (CR) rate was 45% and the partial remission (PR) rate was 5%. The 1-year overall survival (OS) was 56.7% (95% confidence interval (95% CI), 31.6–75.6%), and the median OS was 19 months. The 1-year relapse-free survival (RFS) was 83.3% (95% CI, 27.3–97.5%). Patients relapsing more than 6 months after HSCT and achieving CR/PR after CCAG plus DLI regimen attained significantly higher survival rates. The cumulative incidence of grade III–IV acute graft-versus-host disease (aGVHD) was 9.4%. There was no treatment-related mortality (TRM). These data suggest that CCAG plus DLI regimen is safe and induces durable remission and superior survival in patients with relapsed AML/MDS after allo-HSCT. Trial registration number: ChiCTR.org identifier: ChiCTR1800017740 and date of registration: August 12, 2018.

**Supplementary Information:**

The online version contains supplementary material available at 10.1007/s12032-022-01911-9.

## Introduction

Relapse is the leading cause of treatment failure and mortality after allogeneic hematopoietic stem cell transplantation (allo-HSCT) in hematologic malignancies. The cumulative incidence of relapse after allo-HSCT for acute myeloid leukemia (AML) can be as high as 40–50% with a long-term survival rate of only 10–15% [1–3]. Chemotherapy in combination with donor lymphocyte infusion (DLI) is a standard treatment for relapsed AML/myelodysplastic syndrome (MDS) after allo-HSCT. Unfortunately, the therapy induces remission in less than 40% of patients [4]. Besides, pancytopenia, infection, and graft-versus-host disease (GVHD) frequently occur in patients [5], leading to poor outcomes. Therefore, the best strategy for administering this treatment still needs to be explored.

Increasing evidence suggests that chidamide, a benzamide-class histone deacetylase inhibitor (HDACI), is effective in AML. A pilot study showed that chidamide, fludarabine, and cytarabine combined with granulocyte colony-stimulating factor (G-CSF) (chidamide-FLAG) had a comparable complete remission (CR) rate (57.1%) and lower treatment-related mortality (TRM: 0%) than FLAG plus idarubicin or FLAG plus idarubicin and gemtuzumab ozogamicin (CR: 51%, TRM: 9%) in relapsed/refractory patients [6]. In another study, 24 AML patients with positive minimal residual disease (MRD^+^) after allo-HSCT were administered chidamide and decitabine plus thymalfasin. Strikingly, all patients responded. The overall survival (OS) rate was 79.2%, with a relapse-free survival (RFS) rate of 79.2% [7]. However, the effect of chidamide in patients with frank hematological relapse after HSCT has not been studied. Chidamide not only has a direct cytotoxic effect on tumor cells but can also enhance tumor cell killing by immune cells [8]. Therefore, chidamide might enhance the graft-versus-leukemia (GVL) effect. In this study, we combined chidamide with DLI to treat patients who relapsed after HSCT.

Cytarabine plus aclarubicin and G-CSF (CAG) regimen has been proven successful in refractory and relapsed AML. Several mechanisms for its high efficacy have been proposed: (1) AML cells transition out of the G0 phase because G-CSF stimulation via cell surface receptors allows the other chemical agents to kill resting leukemic cells; (2) Priming with G-CSF effectively enhances cytarabine-induced apoptosis of AML cells; (3) The addition of G-CSF to the regimen enhances the ability of low doses of cytarabine to induce the differentiation of AML cells; (4) Aclarubicin is effective regardless of multi-drug resistance gene status [9]. The CAG regimen also has low toxicity because its doses of cytarabine and aclarubicin are low, and G-CSF shortens the duration of neutrophilia. Although the CAG regimen is effective and has few adverse events (AEs) in the treatment of refractory/relapsed and aged AML/MDS patients, whether it is suitable for treating relapsed disease after HSCT is unknown.

We hypothesized that chidamide plus the CAG regimen (CCAG) followed by DLI might boost the antitumor effect and reduce the toxicity of chemotherapy. Thus, we performed a phase II study to investigate the efficacy and safety of the CCAG regimen with DLI for patients with relapsed AML/MDS after allo-HSCT.

## Methods

### Study design and patient selection

This is a phase II, single-arm clinical trial registered at Chinese PLA General Hospital and with ChiCTR.org identifier ChiCTR1800017740. The trial was approved by the Ethics Committee of Chinese PLA General Hospital and conducted according to the Declaration of Helsinki. Its primary objective was to assess the efficacy and safety of CCAG plus DLI regimen. The study was open for patient accrual from September 2018 to April 2022. Eligible patients were ≥ 18 years old and had relapsed AML or MDS after allo-HSCT, Eastern Cooperative Oncology Group performance status ≤ 3, and expected survival time ≥ 4 months. The exclusion criteria included (1) Patients who were allergic or had contraindications to the drugs; (2) Patients at pregnant or lactation period; (3) Patients with active infection; (4) Patients who smoked or indulged in excessive drinking, affected the outcome of the trial; (5) Patients with mental illness or other conditions could not get informed consent, cooperate with treatment and physical examination; (6) Patients with active bleeding; (7) In the past year, patients had thrombosis, embolism, cerebral hemorrhage, and so on; (8) Patients received surgical treatment, and the operation period was less than 6 weeks; (9) Patients with clinically significant QTC interval prolongation (time of male is more than 450 ms, time of female is more than 470 ms), ventricular tachycardia, atrial fibrillation, and above two degree cardiac block. Patients with myocardial infarction within 1 years, congestive heart failure, coronary artery heart with symptom and needed to be treated. Patients with heart disease, the ultrasound showed that the area of the pericardial cavity was more than 10 mm in the end diastole; (10) Patients with liver dysfunction, total bilirubin > 1.5 times normal upper limit, alanine aminotransferase/aspartate aminotransferase > 2.5 times normal upper limit. Patients with liver involvement, alanine aminotransferase/aspartate aminotransferase > 5 times normal upper limit. Patients with renal dysfunction, peripheral blood creatinine > 1.5 times normal upper limit; (11) Patients with grade II–IV acute GVHD (aGVHD); (12) The researchers decided that participants were not suitable to participate in the clinical trial. All participants gave written informed consent.

### Treatment protocol

Patients received chidamide 30 mg on days 1, 4, 8, and 11. Cytarabine 10 mg/m^2^ was given every 12 hours from days 1 to 5 as preemptive treatment for MRD^+^ patients, and cytarabine 100 mg given every 12 hours from days 1 to 5 as therapeutic treatment for patients with hematologic relapse. Aclarubicin 20 mg was given on days 1, 3, and 5. G-CSF 300 µg was given from day 0 until neutrophils ≥ 2×10^9^/L was achieved. DLI was performed on day 7. Lymphocytes were obtained from cryopreserved G-CSF-mobilized peripheral blood (for G-CSF-primed DLI) or freshly separated peripheral blood (for unprimed DLI). CCAG plus DLI regimen was given every 30 to 45 days and could be stopped when negative MRD (MRD^-^) was detected. The timing of treatment response detection was 1 month after the end of each cycle of treatment. After all treatments, patients had an bone marrow and MRD detection at 1, 2, 3, 4.5, 6, 9, and 12 months and at 6-month intervals thereafter to monitor relapse. The study treatment was designed to be administered for a maximum of 3 consecutive cycles. In the case of grade 3/4 toxicities, CCAG plus DLI regimen was postponed until the toxicities improved. In the case of disease progression, the occurrence of unacceptable toxicities, or withdrawal of consent, the study treatment was discontinued.

### Definitions

Partial remission (PR) of AML was defined as decrease of bone marrow blast percentage to 5 to 25% and decrease of pretreatment bone marrow blast percentage by at least 50%. CR of AML was defined as bone marrow blasts in an aspirate with spicules < 5%, absence of circulating blasts and blasts with Auer rods, absence of extramedullary disease, absolute neutrophil count > 1.0×10^9^/L, and platelet count > 100×10^9^/L [10]. CR of MDS was defined as myeloblasts ≤ 5% with normal maturation of all cell lines, hemoglobin ≥ 11 g/dL, platelet count ≥ 100×10^9^/L, neutrophil ≥ 1.0×10^9^/L, and blasts 0% in peripheral blood. PR of MDS was defined as all CR criteria if abnormal before treatment except: bone marrow blasts decreased by ≥ 50% over pretreatment but still > 5%, and cellularity and morphology not relevant [11]. For MRD detection, we used two strategies for tests in bone marrow samples. MRD^−^ was defined as CR with negativity for a genetic marker (for example, BCR-ABL, AML-ETO, CBFβ-MYH11, and NPM1) by real-time quantitative polymerase chain reaction and CR with negativity by flow cytometry [12]. Hematologic relapse was defined as bone marrow blasts ≥ 5%, reappearance of blasts in the blood or development of extramedullary disease after CR. OS was defined as the length of time from the termination of the first cycle of CCAG plus DLI regimen to death or the last follow-up date. RFS for patients achieving CR was measured from the date of CR documentation to the date of relapse or last follow-up.

### Statistical methods

Descriptive statistics were used for patient characteristics. The cumulative incidences of GVHD were calculated using cumulative incidence curves to accommodate competing risks by using Gray’s method. The Fine-Gray hazards model was used for the univariate analysis of CR/PR and GVHD. Survival analysis was performed by the Kaplan–Meier method, and a Cox proportional hazard model was used to assess the prognostic significance of the clinical variables. A two-sided *P* < 0.05 was considered statistically significant. Statistical analyses were performed with R statistical software (R package 4.1.0) and SPSS 20.0 software.

## Results

### Patient characteristics

Patient characteristics are shown in Table 1 and Table S1. Twenty consecutive patients with relapsed hematologic malignancies after allo-HSCT were enrolled in this study. Diseases included AML (90%) and MDS (10%). The median age was 47.5 years old (range, 21–60 years old), and the majority of patients were male (75%). According to the National Comprehensive Cancer Network (NCCN) guidelines and the revised International Prognostic Scoring System, 35% of patients were classified as intermediate risk, 60% of patients were classified as poor risk, and 5% of patients were classified as very poor risk. Eight patients (40%) were not in remission before transplant. Twelve patients (60%) received HLA-haploidentical-related HSCT. The primary indication for intervention was hematologic relapse (95%), and only 1 patient was MRD^+^. The median percentage of blasts in bone marrow in patients with hematologic relapse was 40.2% (range, 5.6–84.9%), and 70% of patients had a mixed chimerism status at relapse. The interval from HSCT to relapse was 13 months (range, 2–109 months). Six patients (30%) had disease recurrence within 6 months after HSCT, and the other patients (70%) had disease recurrence more than 6 months later.

**Table 1:** Patients’ characteristics

Characteristics	Number (%)
Age, years (median, range)	47.5 (21–60)
Gender of patient	
Male	15 (75)
Female	5 (25)
Gender match with the donor	
Mismatched	10 (50)
Matched	10 (50)
Diagnosis	
AML	18 (90)
MDS	2 (10)
Cytogenetic subgroup ^a^	
Intermediate	7 (35)
Poor	12 (60)
Very poor	1 (5)
No. of induction chemotherapies ^b^	
1	9 (45)
≥ 2	9 (45)
Remission state before HSCT	
CR1	10 (50)
Non-CR1	10 (50)
Donor type	
HLA-identical related	7 (35)
Unrelated	1 (5)
HLA-haploidentical	12 (60)
Percentage of blasts in BM at relapse, % (median, range)	39.4 (2–84.9)
Chimerism status at relapse	
Mixed	14 (70)
Full donor	5 (25)
Unknown	1 (5)
Interval from HSCT to relapse, months (median, range)	13 (2–109)

*AML* acute myeloid leukemia; BM: bone marrow; *CR* complete remission; *HSCT* hematopoietic stem cell transplantation; *MDS* myelodysplastic syndrome.

^a^NCCN guidelines for AML and the revised International Prognostic Scoring System for MDS;

^b^One patient with MDS did not receive any treatment; the other patient with MDS received azacytidine 100 mg for 7 days.

### Treatment response

A total of 30 CCAG plus DLI regimen were given to 20 patients. The median number of interventions per patient was 1 (range, 1–3). Among all patients, 12 (60%) patients received 1 cycle of the protocol treatment, 6 (30%) received 2 cycles of the protocol treatment, and 2 (10%) received 3 cycles of the protocol treatment. The median infused cell doses were 4.9×10^7^ mononuclear cells/kg (range, 0.23–11×10^7^ mononuclear cells/kg), 1.81×10^7^ CD3^+^ cells/kg (range, 0.13–6.04×10^7^ CD3^+^ cells/kg), 0.94×10^7^ CD4^+^ cells/kg (range, 0.08-3.47×10^7^ CD4^+^ cells/kg), and 0.66×10^7^ CD8^+^ cells/kg (range, 0.03-2.75×10^7^ CD8^+^ cells/kg). Fifty percent of patients received unprimed DLI, 45% received G-CSF–primed DLI, and only 1 patient (5%) first received G-CSF–primed DLI and then received unprimed DLI (Table S2). Patient 1 received chemotherapy before CCAG plus DLI regimen, but the interval between chemotherapy and the study treatment was more than a month. At first, patient 12 did not complete CCAG plus DLI regimen as planned because cells from unrelated donors could not be obtained. One month later, he received CCAG again, followed by DLI.

The median follow-up time was 12 months (range, 1–30 months). Among the 20 patients who received CCAG plus DLI regimen, 9 (45%) achieved CR after the first round of protocol treatment. All these patients were MRD^−^ and with full donor chimerism when they completed the treatment. One patient who achieved PR withdrew from the clinical trial and intended to receive a second transplant. Of the other patients who did not achieve CR/PR after CCAG and DLI therapy, 5 patients received other therapies. However, CR was never achieved. Three patients refused further treatment, and 2 had rapidly progressive disease (Table 2). The time from transplantation to relapse was the only possible factor associated with CR/PR (*P* = 0.065, Table 3), with a median time of 18.5 months (range, 2–109 months) in responders and 5 months (range, 2–22 months) in nonresponders (Table 2).

**Table 2:** Outcomes after CCAG plus DLI therapy

Pt.	Remission after CCAG plus DLI regimen	Remission after receiving other therapy ^a^	Remission duration, days	Outcome and cause of death	Follow-up, days
1	CR, MRD^−^	–	527	Died of the second relapse after allo-HSCT	597
2	CR, MRD^−^	–	856	Died of the second relapse after allo-HSCT	939
3	No response	No response	–	Died of relapse	113
4	No response	No	–	Died of relapse	75
5	CR, MRD^−^	–	125	Missing	164
6	CR, MRD^−^	–	334	Survival, extramedullary relapse in 11 months after CR, bone marrow relapse in 20 months after CR	684
7	No response	No	–	Died of relapse	113
8	CR, MRD^−^	–	539	Relapse-free survival	560
9	CR, MRD^−^	–	401	Relapse-free survival	428
10	No response	No response	–	Died of relapse	55
11^b^	CR, MRD^−^	–	358	Relapse-free survival	372
12	No response	No response	–	Died of relapse	158
13	No response	No response	–	Died of relapse	139
14	CR, MRD^−^	–	260	Relapse-free survival	281
15^c^	No response	No	–	Died of relapse	66
16	No response	No	–	Died of relapse	34
17	No response	No	–	Survival, in relapse	175
18	No response	No response	–	Survival, in relapse	162
19	CR, MRD^−^	–	51	Disease-free survival	78
20	PR	No	35	Survival, in PR	60

*CCAG* chidamide, cytarabine, aclarubicin and granulocyte colony-stimulating factor; *CR* complete remission; *DLI* donor lymphocyte infusion; *HSCT* hematopoietic stem cell transplantation; *MRD* negative minimal residual disease; *No* no further treatment; *PR* partial remission; *Pt* patient.

^a^Ten patients did not achieve CR/PR. Five patients received no other treatments. Five patients received other treatments (3-azacytidine/cytarabine/ idarubicin/DLI. 10-chidamide/cytarabine/idarubicin/DLI; azacytidine/cytarabine/etoposide/venetoclax/chidamide/granulocyte colony-stimulating factor; 12-cytarabine; fludarabine/cytarabine/granulocyte colony-stimulating factor/DLI; cytarabine/decitabine; cytarabine/decitabine/chidamide;azacytidine/cytarabine/hydroxyurea.13-cytarabine/idarubicin/decitabine/DLI; cytarabine/etoposide/mitoxantrone/gammadelta T cell infusion; chidamide/azacytidine/cytarabine. 18- cytarabine/idarubicin/DLI).

^b, c^Two patients with myelodysplastic syndrome.

**Table 3:** Univariate analysis for CR/PR and OS after intervention (*n*= 20)

Characteristics	CR/PR		OS	
	OR (95% CI)	*P*	HR (95% CI)	*P*
Age at DLI				
≥ 50 years vs. < 50 years	0.67 (0.21, 2.11)	0.49	1.28 (0.34–4.8)	0.72
Gender of patient				
Male vs. female	0.63 (0.17–2.32)	0.49	1.15 (0.24–5.59)	0.86
Cytogenetic subgroup				
Poor/very poor vs. intermediate	1.79 (0.57–5.69)	0.32	0.83 (0.22–3.1)	0.78
No. of induction chemotherapies				
≥ 2 vs. 1	1.37 (0.41–4.55)	0.61	0.61 (0.14–2.75)	0.52
Remission state before transplant				
Non-Remission vs. remission	0.75 (0.18–3.1)	0.69	0.75 (0.18–3.15)	0.69
Donor type				
HLA-haploidentical related vs. HLA-identical related/unrelated	0.61 (0.19–1.92)	0.4	0.87 (0.23–3.24)	0.83
Gender of donor				
Male vs. female	1.21 (0.32–4.58)	0.77	0.67 (0.18–2.51)	0.56
Gender match with the donor				
Mismatched vs. Matched	2.05 (0.63–6.71)	0.24	0.74 (0.2–2.8)	0.66
Chimerism status at relapse				
Mixed vs. Full donor	3.25 (0.38–27.86)	0.28	0.54 (0.13–2.27)	0.4
Percentage blasts in BM at relapse				
> 20% vs. ≤ 20%	0.79 (0.24–2.54)	0.69	1.57 (0.38–6.46)	0.53
Interval from HSCT to relapse				
≤ 6 months vs. > 6 months	0.17 (0.03–1.12)	*0.065*	4.48 (1.06–18.91)	*0.04*
Cell type				
Unprimed vs. G-CSF–primed DLI	2.09 (0.63–6.93)	0.23	1.18 (0.31–4.41)	0.81

*BM* bone marrow; *CI* confidence interval; *CR* complete remission; *DLI* donor lymphocyte infusion; *G-CSF* granulocyte colony-stimulating factor; *HR* hazard ratio; *HSCT* hematopoietic stem cell transplantation; *OR* odds ratio; *OS* overall survival; *PR* partial remission.

### Survival and RFS

**Fig 1:**
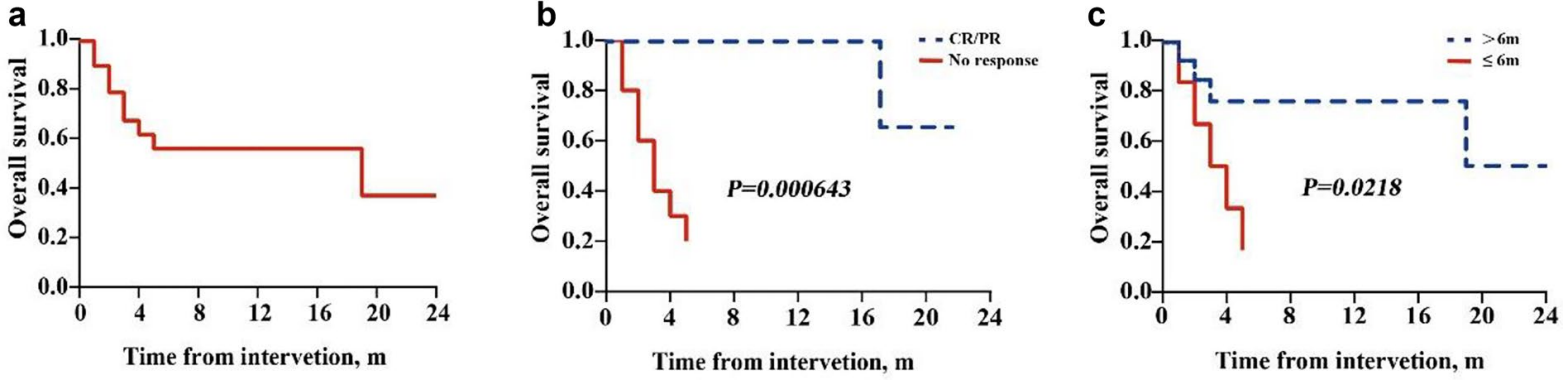
OS of all participants. **a** One-year OS in all participants: 56.7% (95% CI, 31.6–75.6%); the median interval of OS: 19 months. **b** OS in participants with CR/PR vs no response (*P*=0.000643). **c** OS in participants with relapse within 6 months vs more than 6 months after transplantation (*P* = 0.0218). *OS* Overall survival; *CI* confidence interval; *CR* complete remission; *PR* partial remission.

Ten patients died and all died of relapse (Table 2). The median OS of all patients was 19 months. The 1-year OS was 56.7% (95% confidence interval (95% CI), 31.6–75.6%) (Fig 1a). Two possible variables were significantly related to OS: the response after CCAG plus DLI regimen and the interval from transplantation to recurrence. The OS of those with CR/PR after the protocol treatment was significantly higher than that of those with no response (*P* = 0.000643, Fig 1b). Patients who relapsed more than 6 months after HSCT had a much longer survival time than those who relapsed less than 6 months (*P* = 0.0218, Fig 1c). There was no clear effect of cytogenetic subgroup, percentage blasts in bone marrow, or chimerism status at relapse on OS (Table 3).

**Fig 2:**
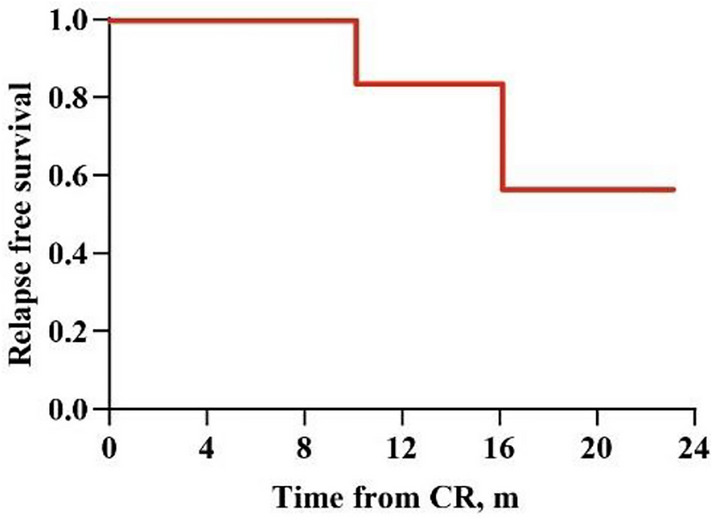
RFS of 9 patients who achieved CR with CCAG plus DLI regimen. One-year RFS: 83.3% (95% CI, 27.3–97.5%). *RFS* relapse-free survival; *CR* complete remission; *CCAG* chidamide, cytarabine, aclarubicin and granulocyte colony-stimulating factor; *DLI* donor lymphocyte infusion; *CI* confidence interval

In 9 patients who achieved CR after CCAG plus DLI regimen, 3 ultimately experienced a second relapse after allo-HSCT. One of them had extramedullary relapse before she had bone marrow relapse. Two of them had bone marrow relapse and died of the second relapse (Table 2). The 1-year RFS was 83.3% (95% CI, 27.3–97.5%, Fig 2).

### GVHD and toxicity

**Fig 3:**
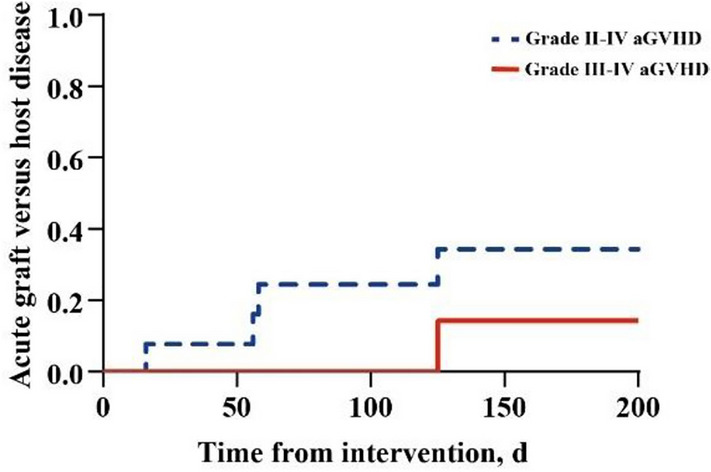
aGVHD of 13 participants who only accepted CCAG plus DLI regimen in a short time. The rate of grade II–IV aGVHD: 33.5; 95% CI, 10–59.5%. The rate of grade III–IV aGVHD: 9.4%; 95% CI, 0.6–34.2%. aGVHD: acute graft-versus-host disease; *CCAG* chidamide, cytarabine, aclarubicin, and granulocyte colony-stimulating factor; *DLI* donor lymphocyte infusion; *CI* confidence interval.

In this study, patients not achieving CR received more intensive chemotherapy and higher-dose DLIs. It has been shown that strong chemotherapy and repeated DLIs in a short time result in GVHD. Therefore, when we assessed aGVHD, we included 13 patients who only received the CCAG plus DLI regimen within several months. The cumulative incidence of grade II–IV aGVHD was 33.5%, with only 9.4% of patients having grade III–IV aGVHD (Fig 3). Of the evaluable patients, 3 patients had grade II aGVHD, and 1 had grade IV aGVHD. Grade IV aGVHD occurred in a patient who received 3 cycles of CCAG plus DLI regimen in three months. No risk factors associated with aGVHD were found (Table S3). cGVHD was noted in 4 (20%) of the 20 enrolled patients: 1 had mild cGVHD with eyes and skin involvement, 2 had moderate cGVHD with eyes and skin involvement, and 1 had severe cGVHD with eyes and lung involvement. All these patients were in a stable condition.

AEs considered to be related to CCAG plus DLI regimen are shown in Table 4 and Fig S1. Hematological AEs were the most common complications. Grade 3–4 neutropenia and thrombocytopenia were noted in all patients. Grade 3–4 leukopenia and anemia were observed in 90 and 80% of patients. The hematological AEs were reversible in responders after CCAG regimen was terminated but not in nonresponders. Grade 3 pneumonitis occurred in 10% of patients, and febrile neutropenia occurred in 15% of patients. All other AEs observed, including gamma-glutamyltransferase increase and alkaline phosphatase increase, were resolved with symptomatic treatment. None of the patients died of AEs.

**Table 4:** Common adverse events of all participants

Adverse events by system organ class	Number of patients (%)	
	All grades	Grade 3–4
Blood and lymphatic system disorders		
Platelet count decreased	20 (100)	20 (100)
Anemia	20 (100)	16 (80)
Neutrophil count decreased	20 (100)	20 (100)
White blood cell decreased	19 (95)	18 (90)
Febrile neutropenia	3 (15)	3 (15)
Respiratory, thoracic and mediastinal disorders		
Cough	5 (25)	5 (25)
Pneumonitis	4 (20)	2 (10)
Gastrointestinal disorders		
Mucositis oral	3 (15)	1 (5)
Nausea	9 (45)	0 (0)
Vomiting	5 (25)	0 (0)
Diarrhea	2 (10)	0 (0)
Metabolism and nutrition disorders		
Anorexia	4 (20)	0 (0)
Hypoalbuminemia	3 (15)	0 (0)
Hyperglycemia	2 (10)	0 (0)
Hypocalcemia	2 (10)	0 (0)
Hypokalemia	2 (10)	0 (0)
Investigations		
Aspartate aminotransferase increased	2 (10)	1 (5)
Alanine aminotransferase increased	5 (25)	2 (10)
Alkaline phosphatase increased	4 (20)	1 (5)
Gamma-glutamyltransferase increased	9 (45)	3 (15)
Blood bilirubin increased	3 (15)	1 (5)
Creatinine increased	2 (10)	0 (0)

## Discussion

Chemotherapy followed by DLI is a promising treatment for relapsed AML/MDS after allo-HSCT. However, the effects of chemotherapy plus DLI are not ideal, and there are many complications. Therefore, we conducted a study using CCAG plus DLI regimen to achieve CR in patients who relapsed after allo-HSCT without causing unacceptable toxicity or GVHD. In this study, the CCAG plus DLI regimen demonstrated a CR of 45%, the 1-year OS of 56.7% (95% CI, 31.6–75.6%), the 1-year RFS of 83.3% (95% CI, 27.3–97.5%), and no treatment-related issues. To the best of our knowledge, this study represents the first prospective clinical trial evaluating the efficacy and safety of CCAG plus DLI regimen in relapsed AML/MDS after allo-HSCT.

In 2004, S-J Choi et al. reported the results of a prospective study evaluating the efficacy of etoposide, cytarabine, idarubicin, and G-CSF followed by G-CSF-primed DLI in patients with advanced myeloid malignancy who relapsed after allo-HSCT [13]. The researchers enrolled 16 AML patients whose disease status at initial HSCT (beyond CR1: 25 vs. 44.4%) and time from initial HSCT to relapse (≤6 months: 37.5 vs. 30%) were comparable with those of our patients. Their therapy had a superior CR rate (62.5 vs. 45%), lower 1-year OS (38 vs. 56.7%), a higher III–IV aGVHD rate (61.5 vs. 9.4%), and an inferior TRM rate (25 vs. 0%) compared with our study. Although we utilized less intensive chemotherapy and a much lower dose of CD3^+^ cells, the CR and OS were comparable to those of the study by Choi et al. We hypothesize that chidamide might play an important role in our treatment: (1) Chidamide directly inhibits the proliferation of human AML cell lines and synergistically enhances apoptosis when combined with cytarabine [14] or low-dose CAG [15] in leukemia cell lines. Our previous study also demonstrated that chidamide increased the sensitivity of anthracycline-resistant cells to anthracycline drugs [16]. As might be expected, CCAG regimen was proved to be effective in treating relapsed AML/MDS after transplantation in our study; (2) Chidamide has a pleiotropic effect on the immune system. Chidamide was shown to enhance the cytotoxic effect of human peripheral blood mononuclear cells (PBMCs) on K562 target cells ex vivo, by upregulating proteins on PBMCs involved in NK-cell functions [8]. Chidamide might also reverse the innate and adaptive immune response deficiencies of PD-1^+^ cells [17]; (3) Chidamide can also regulate the expression of some surface antigens on tumor cells. Chidamide effectively increased the sensitivity of K562 cells to NK cells, which was related to the upregulation of ULBP2 on the surface of K562 cells [18]. Yao et al. showed that chidamide upregulated PRAME and CD86 expression in AML cells and increased PRAME-specific cytotoxic T-lymphocyte killing [19]. Based on these data, we speculate that chidamide simultaneously influences lymphocytes and AML cells and thus enhances the GVL effect. Therefore, chidamide, CAG regimen, and DLI after allo-HSCT have a synergistic effect.

An attractive safety profile of CCAG plus DLI regimen, which showed tolerable and mostly reversible side effects, was also noted in our study. This promising outcome might have been a result of the low toxicity of the CAG regimen and the continuous use of G-CSF. Chemotherapy before DLI has been used for decades. However, most chemotherapy regimens are similar to the high-dose regimens used before transplantation, resulting in 20% TRM of relapsed patients after allo-HSCT in earlier reports [20, 21]. Many of these patients cannot tolerate the intensive chemotherapy protocol because of poor physical condition. Therefore, the low-toxicity treatment would be a better alternative. The CAG regimen is widely used to treat AML/MDS patients, particularly high-risk and elderly patients. A meta-analysis showed that the toxicity of CAG was generally mild, inducing cardiotoxicity in 2.3% and early death in 5.2% of 1029 AML and 215 MDS patients [9]. Our results also support the safety of the CAG regimen, which had a TRM of 0%.

A significantly lower incidence of aGVHD was found in this study. There are several possible reasons for this result: (1) The CAG regimen has lower toxicity and causes minor organ damage; (2) Patients were given immunosuppressive agents to prevent aGVHD; (3) Chidamide might regulate the immune system, leading to less aGVHD. Although there are no studies exploring the effect of chidamide on GVHD, some relevant results can provide some clues. Zhao and his colleagues demonstrated that low-dose chidamide stimulated the production of natural Foxp3^+^ regulatory T (Treg) cells, promoted the peripheral conversion of T cells into Treg cells, and restored Treg cell suppression in vivo and in vitro [22]. Another HDACI, SAHA, was used in a mouse model of bone marrow transplantation (BMT) from day +3 to day +7 after BMT. SAHA reduced serum levels of proinflammatory cytokines, and decreased poor intestinal histopathology findings, clinical severity, and mortality from aGVHD compared with vehicle [23]. In addition, vorinostat increased Treg cell numbers, upregulated the expression of CD45RA and CD31 on Treg cells [24], and downregulated IL-6 and GVHD biomarkers, including soluble ST2 and Reg3a, resulting in a low incidence of aGVHD [25]. Further research of the relationship of chidamide and GVHD is needed.

We further analyzed the factors related to CR/PR and OS and found much worse outcomes in patients who relapsed within less than 6 months after allo-HSCT. This result is in accordance with previous studies, in which early relapse heralded a dismal prognosis [20, 26]. Patients who relapse early after BMT may represent a subset of patients with chemotherapy-resistant disease, and more intensive chemotherapy is needed to reduce tumor burden. Other mechanisms of relapse include genomic loss of HLA [27], transcriptional downregulation of HLA class II molecules [28, 29], and the enforcement of inhibitory checkpoints between T cells and leukemia cells [30]. Genomic loss of HLA cannot be reversed; however, the expression of HLA class II molecules and inhibitory checkpoints can be regulated by epigenetic therapies. HDACIs, such as vorinostat [31] and panobinostat [24], have been associated with the upregulation of major histocompatibility and costimulatory molecules on the AML cell surface through the induction of an open and readable structure of chromatin. Further study of HLA class II molecules and inhibitory checkpoints on tumor cells before and after chidamide treatment is needed.

This study has several limitations. First, our data should be interpreted cautiously considering the limited number of patients. Second, in this study, there was a male predominance of the population, which may have induced sampling bias and limit the applicability of the results to all patients. Some articles have shown that the treatment effect of chemotherapy and DLI in females is better than that in males [21]. Therefore, the CR rate and efficacy in this paper may be underestimated. Third, we did not examine HLA class II molecules and inhibitory checkpoints on tumor cells of the enrolled patients before and after chidamide treatment. However, an in vitro experiment is under way.

In conclusion, CCAG plus DLI regimen was feasible and effective in relapsed AML/MDS patients after allo-HSCT, especially in patients with a relatively long post-HSCT remission duration. A study with a larger cohort is needed to verify the results.

## Supplementary Information

Below is the link to the electronic supplementary material.

Supplementary file1 (DOCX 336 KB)

## Data Availability

The data that support the findings of this study are available on request from the corresponding author.
