# Supplementary material for: A phase II study of chidamide, cytarabine, aclarubicin, granulocyte colony-stimulating factor, and donor lymphocyte infusion for relapsed acute myeloid leukemia and myelodysplastic syndrome after allogeneic hematopoietic stem cell transplantation
Source: Med Oncol. 2023 Jan 10;40(2):77. doi: 10.1007/s12032-022-01911-9 (PMC9832090; doi:10.1007/s12032-022-01911-9)
Supplement: Supplementary file 1 — Supplementary file1 (DOCX 336 KB) [file 12032_2022_1911_MOESM1_ESM.docx]

**Supplement****ary**

| Pt. | Age/Gender | Diagnosis | Cytogenetic subgroup | No. of induction chemotherapies | Remission state before HSCT | Donor type | Donor | Indication for intervention | Percentage of blasts in BM at  relapse, % | Chimerism status at relapse | Interval from HSCT to relapse, months |
| --- | --- | --- | --- | --- | --- | --- | --- | --- | --- | --- | --- |
| 1 | 51/M | AML | Intermediate | 1 | CR1 | HLA-identical related | Brother | Therapeutic | 14 | Mixed | 109 |
| 2 | 36/M | AML | Poor | 1 | CR1 | HLA-haploidentical related | Sister | Therapeutic | 6.4 | ─ | 17 |
| 3 | 30/F | AML | Poor | 1 | CR1 | HLA-haploidentical related | Sister | Therapeutic | 77.5 | Mixed | 11 |
| 4 | 47/M | AML | Intermediate | 1 | Relapse | HLA-identical related | Sister | Therapeutic | 48 | Mixed | 3 |
| 5 | 54/M | AML | Intermediate | 2 | CR1 | HLA-identical related | Brother | Therapeutic | 55.2 | Mixed | 2 |
| 6 | 53/F | AML | Poor | 1 | CR1 | HLA-haploidentical related | Son | Therapeutic | 38.8 | Mixed | 12 |
| 7 | 52/F | AML | Intermediate | 1 | CR1 | HLA-identical related | Brother | Therapeutic | 32.8 | Mixed | 3 |
| 8 | 48/M | AML | Poor | 2 | Relapse | HLA-identical related | Sister | Therapeutic | 48.8 | Mixed | 18 |
| 9 | 39/M | AML | Intermediate | 2 | CR2 | HLA-haploidentical related | Sister | Therapeutic | 19.5 | Mixed | 34 |
| 10 | 50/M | AML | Poor | 1 | CR2 | HLA-haploidentical related | Brother | Pre-emptive | 2 | Full donor | 2 |
| 11 | 56/F | MDS-RAEB-1 | Poor | Azacitidine | MDS-RAEB-1 | HLA-identical related | Brother | Therapeutic | 8 | Full donor | 19 |
| 12 | 22/M | AML | Intermediate | 2 | CR1 | Unrelated | Unrelated donor | Therapeutic | 84.9 | Mixed | 3 |
| 13 | 51/M | AML | Poor | No response | No response | HLA-haploidentical related | Son | Therapeutic | 5.6 | Full donor | 3 |
| 14 | 35/M | AML | Poor | No response | No response | HLA-haploidentical related | Father | Therapeutic | 40.8 | Mixed | 22 |
| 15 | 60/M | MDS-RS | Very poor | 0 | MDS-RS | HLA-haploidentical related | Son | Therapeutic | 40 | Full donor | 22 |
| 16 | 39/M | AML | Poor | 2 | CR1 | HLA-haploidentical related | Sister | Therapeutic | 64.8 | Mixed | 14 |
| 17 | 58/M | AML | Poor | No response | No response | HLA-haploidentical related | Son | Therapeutic | 46.4 | Mixed | 10 |
| 18 ^a^ | 21/M | AML | Intermediate | 1 | No response | HLA-haploidentical related | Brother | Therapeutic | 6 | Full donor | 7 |
| 19 | 34/M | AML | Poor | 2 | CR1 | HLA-haploidentical | Father | Therapeutic | 28.5 | Mixed | 61 |
| 20 | 47/F | AML | Poor | 2 | CR1 | HLA-identical related | Brother | Therapeutic | 42.4 | Mixed | 18 |

**Table S1** Individual patient and disease characteristics at baseline

AML: acute myeloid leukemia; BM: bone marrow; CR: complete remission; F: female; HSCT：hematopoietic stem cell transplantation; M: male; MDS: myelodysplastic syndrome; Pt: patient; RAEB: refractory anemia with excess blasts; RS: ringed sideroblasts.

^a^ Patient 18 relapsed after a synegenic hematopoietic stem cell transplantation and an [allogeneic hematopoietic stem cell transplantation](javascript:;).

**Table S2** The treatment after relapse and cells infused for CCAG plus DLI regimen

| Pt. | Treatments before intervention | Interval from relapse to intervention, days | Cell type of DLI | MNC, 10^7^/Kg | CD3^+^ cells, 10^7^/Kg | CD3^+^CD4^+^ cells, 10^7^/Kg | CD3^+^CD8^+^, cells, 10^7^/Kg | GVHD prophylaxis |
| --- | --- | --- | --- | --- | --- | --- | --- | --- |
| 1 | IA、DA | 189 | Unprimed | 6.32 | 3.70 | 1.98 | 1.45 | MTX |
|  | Chidamide+IA | 398 | Unprimed | 4.99 | 2.35 | 1.21 | 0.97 | MTX |
| 2 | No | 22 | G-CSF–primed | 5.00 | 2.05 | 0.94 | 0.86 | MTX |
| 3 | hydroxyurea | 8 | G-CSF–primed | 11.00 | 6.04 | 3.47 | 1.89 | CSA |
| 4 | No | 11 | G-CSF–primed | 9.82 | 4.27 | 2.90 | 1.19 | MTX |
| 5 | No | 43 | G-CSF–primed | 5.50 | 2.25 | 1.44 | 0.74 | MTX |
|  | No | 100 | G-CSF–primed | 5.00 | 1.71 | 0.99 | 0.66 | MTX |
| 6 | No | 76 | G-CSF–primed | 5.30 | 2.33 | 0.57 | 1.52 | CSA |
|  | No | 140 | G-CSF–primed | 8.80 | 4.24 | 1.09 | 2.75 | CSA |
| 7 | No | 14 | G-CSF–primed | 6.60 | 1.81 | 0.88 | 0.85 | MTX |
| 8 | No | 4 | G-CSF–primed | 9.36 | 3.28 | 1.07 | 0.71 | MTX |
|  | No | 45 | Unprimed | 4.90 | 2.54 | 1.59 | 0.65 | MTX |
|  | No | 88 | Unprimed | 5.00 | 2.46 | 1.59 | 0.60 | MTX |
| 9 | No | 11 | Unprimed | 2.10 | 1.01 | 0.68 | 0.25 | CSA |
| 10 | No | 25 | G-CSF–primed | 5.00 | 2.64 | 1.43 | 1.07 | MTX |
| 11 | No | 2 | Unprimed | 4.90 | 1.83 | 1.07 | 0.61 | MTX |
| 12 | CCAG | 80 | Unprimed | 0.76 | 0.38 | 0.19 | 0.16 | No |
| 13 | No | 15 | G-CSF–primed | 1.20 | 0.36 | 0.19 | 0.12 | CSA |
|  | No | 48 | G-CSF–primed | 9.50 | 3.71 | 2.03 | 1.24 | CSA |
| 14 | No | 24 | Unprimed | 1.00 | 0.17 | 0.14 | 0.03 | MTX |
|  | No | 173 | Unprimed | 2.50 | 0.40 | 0.28 | 0.08 | Sirolimus |
|  | No | 229 | Unprimed | 4.46 | 0.37 | 0.07 | 0.03 | Sirolimus |
| 15 | No | 44 | Unprimed | 1.00 | 0.41 | 0.14 | 0.20 | CSA |
| 16 | No | 35 | Unprimed | 0.23 | 0.13 | 0.08 | 0.04 | CSA |
| 17 | No | 28 | Unprimed | 0.97 | 0.40 | 0.18 | 0.21 | CSA |
| 18 | No | 3 | G-CSF–primed | 2.30 | 0.69 | 0.36 | 0.30 | CSA |
| 19 | No | 16 | Unprimed | 1.00 | 0.47 | - | - | MTX |
|  | No | 68 | Unprimed | 2.00 | 0.94 | - | - | MTX |
| 20 | No | 4 | Unprimed | 2.82 | 1.16 | 0.45 | 0.28 | MTX |
|  | No | 53 | Unprimed | 5.50 | 2.43 | 1.14 | 0.82 | MTX |

CCAG: chidamide, cytarabine, aclarubicin and granulocyte colony-stimulating factor; CSA: cyclosporine A; DA: daunorubicin and cytarabine; DLI: donor lymphocyte infusion; G-CSF: granulocyte colony-stimulating factor; IA: idarubicin and cytarabine; MNC: mononuclear cell; MTX: methotrexate; Pt: patient.

**Table S3** Univariate analysis for grade II-IV aGVHD after CCAG plus DLI regimen (n = 13)

| Characteristics | Grade Ⅱ-Ⅳ aGVHD | |
| --- | --- | --- |
|  | OR (95% CI) *P* | |
| **Age at DLI**  ≥ 48 years vs. < 48 years | 0.3 (0.03-2.98) | 0.3 |
| **Donor type**  HLA-haploidentical related vs. HLA-identical related/Unrelated | 3.61 (0.53-24.72) | 0.19 |
| **Gender of donor**  Female vs. Male | 0.51 (0.09-2.84) | 0.44 |
| **Gender match with the donor**  Mismatched vs. Matched | 1.01 (0.16-6.2) | 0.99 |
| **Chimerism status at relapse**  Mixed vs. Full donor | 0.45 (0.05-3.9) | 0.47 |
| **Interval from HSCT to relapse**  ≤ 6 months vs. > 6 months | 0.69 (0.07-6.45) | 0.75 |
| **Cell type**  Unprimed vs. G-CSF–primed DLI | 1.07 (0.19-6.17) | 0.94 |

aGVHD: acute graft-versus-host disease; CCAG: chidamide, cytarabine, aclarubicin and granulocyte colony-stimulating factor; CI: confidence interval; DLI: donor lymphocyte infusion; G-CSF: granulocyte colony-stimulating factor; OR: odds ratio.


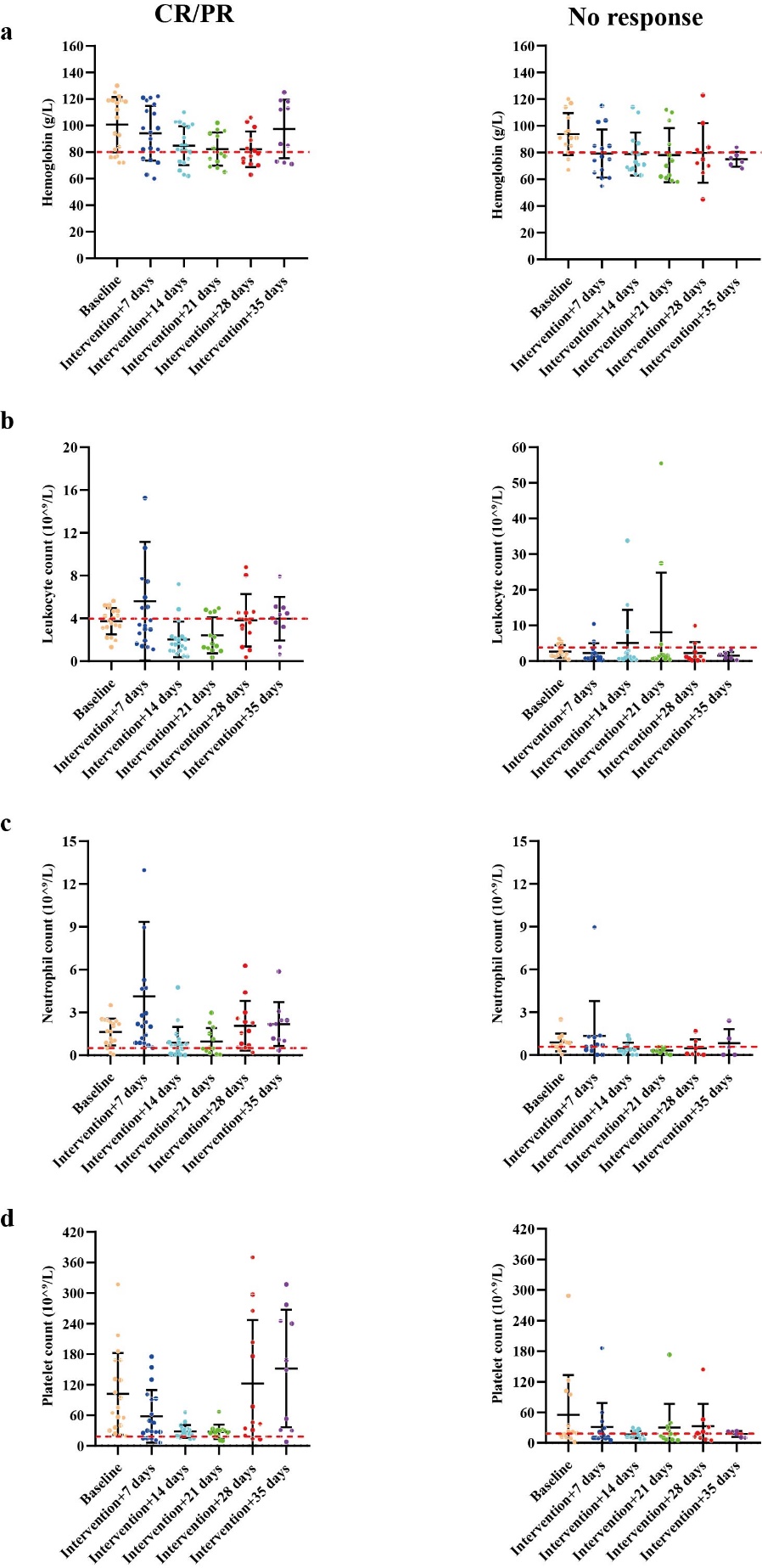


**Fig S1** Hemoglobin, leukocyte, neutrophil and platelet of CR/PR patients and non-response patients at different times after CCAG plus DLI regimen. (a), Red line: 80 g/L. (b), Red line: 4×10^9/L. (c), Red line: 0.5×10^9/L. (d), Red line: 20×10^9/L. CCAG: chidamide, cytarabine, aclarubicin and granulocyte colony-stimulating factor; CR: complete remission; DLI: donor lymphocyte infusion; PR: partial remission.
